# Precision Psychiatry and the Clinical Meaning of Waiting

**DOI:** 10.7759/cureus.104319

**Published:** 2026-02-26

**Authors:** Esteban Zavaleta-Monestel, Jeaustin Mora-Jiménez, Sebastián Arguedas-Chacón, Ricardo Millán González

**Affiliations:** 1 Department of Pharmacy, Hospital Clínica Bíblica, San José, CRI; 2 Department of Research, Hospital Clínica Bíblica, San José, CRI; 3 School of Medicine, Universidad de Costa Rica, San José, CRI

**Keywords:** clinical decision-making, clinical inertia, precision psychiatry, treatment continuation, treatment timing

## Abstract

Psychiatric practice is characterized by uncertainty in diagnosis, prognosis, and treatment response, making waiting a common and often prudent clinical strategy. Despite its central role in everyday care, the clinical meaning of waiting is rarely examined explicitly. This editorial argues that waiting is not the absence of decision-making but an active clinical choice with consequences for symptoms, functioning, and patient experience when treatment continuation becomes the default. Precision psychiatry is often framed around baseline prediction, yet precision also depends on how clinicians manage time, reassessment, and evolving clinical information. Viewing waiting as a time-explicit and revisitable decision emphasizes the importance of predefined reassessment points, transparent expectations, and alignment with patient values and tolerance for uncertainty. Treating waiting as a deliberate decision offers a practical way to enhance precision in routine psychiatric care without sacrificing caution.

## Editorial

Psychiatric practice is defined by uncertainty. Diagnostic categories encompass a wide range of presentations, illness trajectories vary substantially, and treatment effects are often modest and unpredictable. As a result, psychiatric training has long emphasized patience: allowing interventions time to work, tolerating ambiguity, and avoiding premature changes that might destabilize care. This approach is widely regarded as prudent and clinically responsible. Yet despite the centrality of waiting in everyday psychiatric practice, the clinical meaning of waiting itself is rarely examined.

In routine psychiatric practice, waiting is often regarded as part of good clinical care. Few, however, pause to consider what waiting means for patients who continue treatment without improvement, particularly when they are uncertain whether improvement is still expected or whether alternative strategies are being actively considered.

The concern here is not with thoughtful watchful waiting, nor with delays imposed by structural constraints such as limited visit availability, insurance authorization requirements, or workforce shortages. Rather, it lies in an unexamined default that often goes unnoticed in routine care: the assumption that continuing an unchanged treatment requires less justification than altering it. In real-world practice, operational pressures, including limited consultation time, workload demands, and service constraints, may also contribute to this pattern of default continuation. When continuation is treated as the prudent baseline rather than as an active clinical choice, its consequences for symptoms, functioning, and patient experience can become invisible, even as patients experience them day to day.

In routine practice, waiting does not represent the absence of decision-making. It is a decision with tangible consequences. Continuing treatment reflects a judgment that the expected benefits of additional time outweigh the costs of ongoing symptoms, side effects, and uncertainty. Framed this way, the central issue is no longer whether waiting occurs, but how decisions to wait are justified, revisited, and communicated over time. Similar dynamics have been described in other areas of medicine under the concept of clinical inertia, in which treatment persists by default despite signals that reconsideration may be warranted [1].

**Figure 1:**
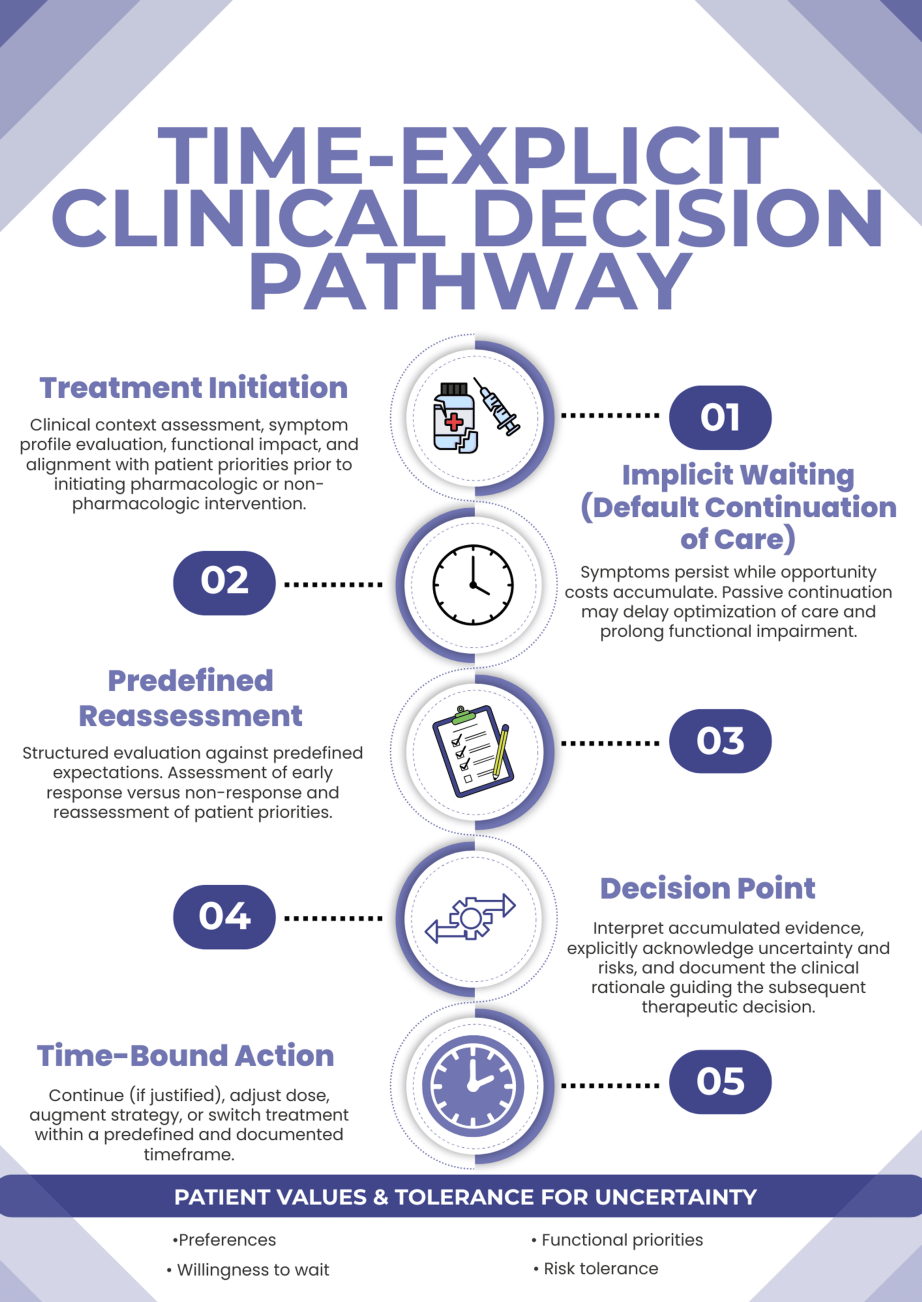
Time-explicit clinical decision pathway The conceptual framework is informed by the literature on clinical inertia and precision psychiatry [1,2]. This figure was created by the authors using Canva Pro (Canva Pty Ltd., Sydney, Australia).

To make this perspective explicit, Figure 1 presents a time-explicit clinical decision pathway for precision psychiatry. Rather than treating waiting as a neutral interval, the framework conceptualizes continuation as an active and revisitable clinical choice. Decisions unfold across predefined reassessment points and are informed by observed clinical response, evolving patient priorities, and tolerance for uncertainty [1,2].

This distinction helps clarify the difference between deliberate watchful waiting and default continuation. Thoughtful waiting is intentional and time-limited. It involves clear expectations, active monitoring, and readiness to adjust care if anticipated improvement does not occur. Much of everyday practice, however, falls somewhere in between. Treatment may continue not because it has been actively chosen, but because changing it would require an explicit decision. In such cases, delay becomes clinically meaningful precisely because it remains unexamined.

Precision psychiatry is often proposed as a response to uncertainty in psychiatric care, typically framed in terms of improved baseline prediction. Biomarkers, neuroimaging, and computational approaches aim to guide treatment selection before interventions begin. While these efforts are scientifically valuable, they risk narrowing the practical meaning of precision. Commentators have cautioned that personalization based solely on baseline prediction may overpromise while neglecting the evolving clinical context in which decisions are made [2]. At the same time, operational pressures such as limited time, workload demands, and service constraints may influence how continuation decisions unfold in practice, shaping how precision is enacted over time.

From a clinical perspective, precision is not limited to choosing an initial treatment. It also concerns determining whether, when, and why to adapt care as new information emerges. Decisions about continuation, therefore, occupy a central role in precision-oriented practice. They reflect judgments about timing, tolerance for uncertainty, and the trade-offs that patients are willing or unwilling to accept. Precision psychiatry, in this sense, extends beyond prediction at baseline to include responsiveness over time.

Prolonged continuation of ineffective treatment carries practical costs. Symptoms may persist, functional impairment may continue, and side effects may accumulate without clear benefit. These effects compound over time and are experienced directly by patients, who live with treatment as an ongoing condition rather than as a series of abstract probabilities. The magnitude and timing of these effects may vary across diagnostic categories, illness trajectories, and treatment modalities, but the clinical burden of prolonged uncertainty remains significant. Uncertainty itself can become burdensome, particularly when patients are unsure whether improvement is still expected or whether alternatives are being considered.

Clinical evidence underscores the importance of timing. In antidepressant trials, lack of meaningful improvement within the first several weeks is associated with a lower likelihood of later remission, although a minority of patients do benefit from extended treatment [3]. Comparable patterns have been observed in structured psychotherapies, where early symptom change predicts longer-term outcomes well before protocol completion [4]. These findings do not mandate early switching, but they challenge the assumption that waiting is neutral or without cost.

Importantly, the clinical meaning of waiting varies across patients. Two individuals with similar symptom severity may experience ongoing treatment very differently. One may prefer to continue despite uncertainty, valuing stability and minimizing change. Another may find ongoing impairment intolerable, particularly when it interferes with work, caregiving responsibilities, or future planning. Life context shapes how patients experience time, and these differences are central to clinical decision-making.

Despite this, discussions about treatment often focus on which intervention to use rather than how long to persist with it. From a shared decision-making perspective, expectations about timing and reassessment should be made explicit rather than assumed. Expectations about timing are frequently implicit. Patients may not know when improvement is anticipated or what will occur if it does not happen. When waiting is not discussed explicitly, it can feel indefinite, even when clinicians believe they are following reasonable timelines.

Treating waiting as a clinical decision brings these issues into the open. It encourages clinicians to articulate expectations, define reassessment points, and clarify what would prompt reconsideration. From a practical standpoint, this approach requires explicit timelines for expected improvement, predefined moments for reassessment, and transparent discussion with patients about what would trigger a change in strategy. These steps do not accelerate care indiscriminately, but they help prevent inertia from being mistaken for prudence. Such conversations align closely with shared decision-making approaches, which emphasize that preference-sensitive choices should be discussed explicitly rather than assumed [5].

This perspective also helps distinguish thoughtful waiting from clinical inertia. Thoughtful waiting is characterized by intention and review. Clinical inertia arises when treatment persists simply because changing course feels more difficult or uncertain. In psychiatry, where delayed response is common, inertia can be difficult to recognize without explicit reassessment. Recognizing waiting as a decision does not imply that change should occur more quickly, but that continuation should be supported by a clear rationale that is revisited over time.

There are many situations in which waiting is clearly appropriate. Early modification of antidepressants may increase risk in patients with possible bipolar spectrum illness. Apparent nonresponse may reflect inadequate dosing, poor adherence, or unresolved psychosocial stressors rather than true treatment failure. In psychotherapy, early distress or symptom fluctuation may precede meaningful improvement. In these contexts, waiting is not passive; it is protective.

Much of the information needed to support more responsive care is already available in routine practice. Measurement-based care models demonstrate that systematic monitoring can modestly improve outcomes, yet their impact depends on how clinicians respond to the information collected [6]. Similarly, stepped-care approaches structure treatment sequencing but often leave the timing of reassessment underspecified, allowing default continuation to persist [7].

Precision psychiatry is often portrayed as a future aspiration dependent on scientific breakthroughs. Yet one of its most immediate contributions lies in how clinicians think about time in everyday practice. Waiting is one of the most common clinical strategies in psychiatry, but it is rarely treated as a decision that requires justification. By making continuation explicit, revisitable, and aligned with patient priorities, psychiatric care can become more precise without sacrificing caution.

Uncertainty will remain a defining feature of psychiatric practice. Precision does not eliminate uncertainty, but it can clarify how uncertainty is managed. Treating waiting as a decision rather than a neutral interval offers a practical way to improve care today by making time itself a visible and deliberate element of clinical practice.
